# Inequities in incidence, morbidity and expenditures on prevention and treatment of malaria in southeast Nigeria

**DOI:** 10.1186/1472-698X-9-21

**Published:** 2009-09-05

**Authors:** Nkolika P Uguru, Obinna E Onwujekwe, Benjamin S Uzochukwu, Godwin C Igiliegbe, Soludo B Eze

**Affiliations:** 1 Department of Preventive Dentistry, Faculty of Dentistry, College of Medicine, University of Nigeria, Enugu, Nigeria; 2 Department of Health Administration and Management, Faculty of Health Sciences and Technology, College of Medicine, University of Nigeria, Enugu, Nigeria; 3 Department of Community Medicine, Faculty of Medical Sciences, College of Medicine, University of Nigeria, Enugu, Nigeria; 4 Malaria and Vector Control Unit, Enugu State Ministry of Health, Enugu, Nigeria; 5 Health Policy Research Group, Department of Pharmacology and Therapeutics, College of Medicine, University of Nigeria, Enugu, Nigeria

## Abstract

**Background:**

Malaria places a great burden on households, but the extent to which this is tilted against the poor is unclear. However, the knowledge of the level of the burden of malaria amongst different population groups is vital for ensuring equitable control of malaria. This paper examined the inequities in occurrence, economic burden, prevention and treatment of malaria.

**Methods:**

The study was undertaken in four malaria endemic villages in Enugu state, southeast Nigeria. Data was collected using interviewer-administered questionnaires. An asset-based index was used to categorize the households into socio-economic status (SES) quartiles: least poor; poor; very poor; and most poor. Chi-square analysis was used to determine the statistical significance of the SES differences in incidence, length of illness, ownership of treated nets, expenditures on treatment and prevention.

**Results:**

All the SES quartiles had equal exposure to malaria. The pattern of health seeking for all the SES groups was almost similar, but in one of the villages the most poor, very poor and poor significantly used the services of patent medicine vendors and the least poor visited hospitals. The cost of treating malaria was similar across the SES quartiles. The average expenditure to treat an episode of malaria ranged from as low as 131 Naira ($1.09) to as high as 348 Naira ($2.9), while the transportation expenditure to receive treatment ranged from 26 Naira to 46 Naira (both less than $1). The level of expenditure to prevent malaria was low in the four villages, with less than 5% owning untreated nets and 10.4% with insecticide treated nets.

**Conclusion:**

Malaria constitutes a burden to all SES groups, though the poorer socio-economic groups were more affected, because a greater proportion of their financial resources compared to their income are spent on treating the disease. The expenditures to treat malaria by the poorest households could lead to catastrophic health expenditures. Effective pro-payment health financing and health delivery methods for the treatment and prevention of malaria are needed to decrease the burden of the disease to the most-poor people.

## Background

About 40% of the world's population, mostly those living in the poorest countries, are at risk of contracting malaria, and of these 2.5 billion people at risk, more than 500 million become severely ill with malaria every year and more than 1 million die from the effects of this disease,[1] which is both preventable and curable.

This disease which is endemic in most African countries constitutes one of the major public health challenges eroding development in the poorest countries in the world [2,3]. It is a serious problem in Africa, where one in every five (20%) childhood deaths is due to its effects [1]. An African child on the average suffers between 1.6 and 5.4 episodes of malaria fever each year and every 30 seconds a child dies from it [1].

Malaria costs Africa more than US$ 12 billion annually and has slowed economic growth in African countries by 1.3% per year while malaria-free countries average three times higher GDP per person [2-4], thus widening the prosperity gap between countries with and those without malaria [3]. In countries with a high prevalence of malaria, the disease may account for as much as 40% of public health expenditures, 30% to 50% of inpatient admissions and up to 50% of outpatient visits [5].

Nigeria accounts for a quarter of all malaria cases in the WHO African Region. Transmission in the south occurs all-year round, and is more seasonal in the north. Almost all cases are caused by *P. falciparum *although most of them are usually unconfirmed [6]. In Nigeria, the National Malaria Control Program delivered about 17 million Insecticide treated bed nets during 2005-2007, enough to cover only 23% of the population at risk. The programme delivered 4.5 million courses of ACT in 2006 and 9 million in 2007 which is far below the country's total requirements [6].

The burden of malaria traps families and communities in a downward spiral of poverty, disproportionately affecting marginalized and poor people who cannot afford treatment or who have limited access to health care [4]. It has lifelong effects through increased poverty and impaired learning [7] and usually reduces attendance at schools and workplaces [8,4]. Thus, potential earnings and household food security are reduced due to frequent illness and malaria deaths leaving millions of households to bear the burden of health expenditures associated with malaria. [4].

The enhanced control of malaria will significantly increase the continent's economic productivity and the income of African families [2]. Hence, in order to achieve the Roll Back Malaria goal of halving the burden of malaria in Africa by 2010, [9,10] there has been an increase in funding for the control of malaria [6]. The increased funding has helped to improve access to malaria control interventions such as insecticide treated bed nets, where the coverage has increased almost eightfold, from 3% in 2001 to 23% in 2006 in eighteen African countries [6]. However, reported US$ 4.6 available per (estimated) malaria case in the 26 reporting countries is unlikely to be adequate to meet targets for prevention and cure [6]

In Nigeria, funding for malaria control which was provided by the government and donors increased from US$ 17 million in 2005 to US$ 60 million in 2007 but this is insufficient to reach national targets for prevention and cure and thus there is no evidence of a systematic decline in malaria burden in Nigeria [6]. Studies have shown that between 2001 and 2007, there was an increase in numbers of malaria deaths from 4,317 in 2001 to 10,289 in 2007 for all ages and 721 to 2,695 for under five year olds, although this upward trend may be due to improvements in reporting of cases [6] as a study showed that high mortality and morbidity rates are on the decline in some African countries [11]. Eritrea, Rwanda and Sao Tome and Principe have reported dramatic reductions in malaria deaths by 50% or more between the years 2000 and 2007 through a mix of bed net distribution, indoor spraying, improved access to treatment and advances in disease surveillance [6].

Spending for malaria can absorb the majority or entire household budget for health, especially of the poor [4,12]. It has been shown that malaria places significant burdens on households that have a sick family member [13-22]. These include time lost from work by the sick individual, care-giving time spent by other family members, lost productivity, costs of seeking treatment (including transportation and medical care), and premature mortality [13,23]. The costs of treating malaria fall particularly heavily on the poor because the direct and indirect costs of a single case often represent a significant portion of a person's income [13]. It was found that the costs of malaria prevention and treatment, added to the foregone income from adult morbidity and caretaking for children with the disease, represent about 20% of annual income in Malawi [24]. Studies in Kenya and Nigeria showed that the lower income households and rural farmers were the hardest hit by malaria's economic impact where the burden of health care costs for malaria in farm households in Kenya and lower income urban house holds in Nigeria are 9% and 13% respectively of annual household incomes [3]. Also, significantly, the higher cost of malaria episodes on the poor is exacerbated by their lower expenditures on and use of malaria preventive tools such as insecticide-treated nets [5].

Some studies have argued that the evidence on the magnitude of the burden of malaria in Nigeria is limited and their value for generalization is also limited due to a limited scope [25]. There is also paucity of evidence in Nigeria and in sub-Saharan Africa about the inequity in malaria occurrence. Previous studies reported that incidence of malaria is typically lower at the very top of the wealth distribution, but the relationship is not strong after controlling for confounding factors [7]. Thus the magnitude and inequity in burden of malaria requires more evidence that will motivate the policy makers to increase resource allocation to malaria control and at the same time ensuring the equitable deployment of such resources.

Hence, this paper contributes to knowledge about the level of inequities in burden of malaria and expenditures on its control in Nigeria, with relevance to other malaria-endemic countries in sub-Saharan Africa. There is the need to build the evidence base on socio-economic inequity in burden of malaria as well as in expenditures to treat and prevent it, which would act as a catalyst to improve equitable policies and strategies for the control of the disease. Inequity has been defined as the presence of unjust but redeemable inequalities [26]. The existence of inequities in the burden of the disease could lead to the poor incurring catastrophic costs that could lead to a deeper state of poverty [4].

## Methods

### Study area

**Figure 1:**
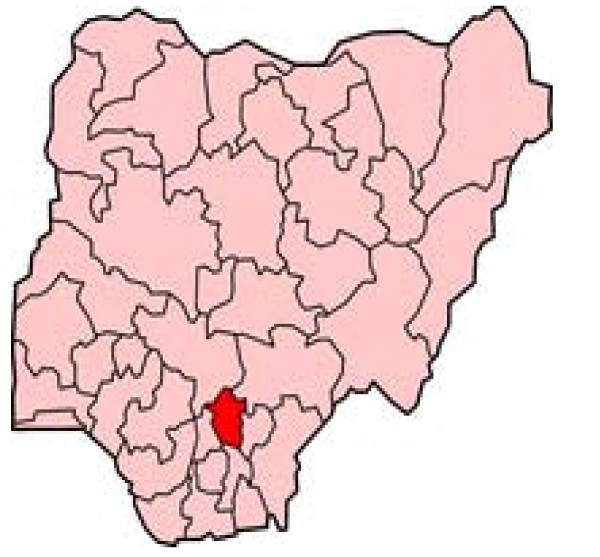
**Map of Nigeria showing Enugu State (in red)**. http://www.nigeriagalleria.com/Nigeria/States_Nigeria/Enugu_State.html

The study took place in four malaria holo-endemic villages in Achi community, Oji-River local government area of Enugu state, Southeast Nigeria as shown in figure 1. Achi community is located 5 kilometers from the local government headquarters called Oji-River and 45 kilometers from the state capital, Enugu. It has an estimated population of 45,000 people and is divided into 12 villages. Achi is linked to Oji-River by a single lane road covered with asphalt, which presents a formidable challenge for users especially during the raining season because the road is covered with potholes. Dirt roads and bush paths provide means of access to the interiors in the villages.

There is a public general hospital and 2 health centers in the town. Three private hospitals/clinics and two maternity homes complement the public providers. There are a number of patent medicine stores in each of the study communities and itinerant drug providers also visit the community on the major market days. Numerous herbalists and other unorthodox healthcare providers (not using western medicine) abound.

### Study design

It was a cross-sectional study using pre-tested interviewer-administered questionnaires to interview the householders. A month recall period was used for the questioning regarding health seeking pattern of the adults and children (less than 13 years) in their households. Data was collected on recent household experiences of presumptive malaria (using presence of fever as a proxy) for respondents and children. Data was also collected on cost of treatment and transportation. The respondents were asked how much they spent on registration, consultation, investigations and drugs. The expenditures on these items were then aggregated to give the total treatment expenditure. Respondents were also asked about the average monthly household expenditures on different types of malaria preventive tools (mosquito nets, insecticide coils and sprays) and any other money spent in the month for the prevention of malaria. Data was also collected on household's ownership of both untreated and insecticide-treated mosquito nets (ITNs).

The sampling frame for the study was developed by an enumeration of the households in the four villages. The numbers of enumerated households were 1,100 in Ahani, 580 in Adu, 750 in Amaetiti and 750 in Enugu-Akwu. Using the formula for sample size for a definite population; considering 0.25 as the proportion of the population positive for malaria and 0.05 as the absolute sampling error that can be tolerated, 280 households was the sample size per village. However, in order to control for non-responders, 300 households were selected from each village using simple random sampling. The respondents were the heads of households or their representatives (where the head was absent). The interviewers were secondary school leavers who were trained for 5 days on data collection techniques.

### Data analysis

Tabulations, equity analysis, testing of means and non-parametric tests were the major data analytic procedures that were used. The data was analyzed individually for each village. The results from each village were individually compared across socio-economic status (SES) groups within each village. However, a pooled data from the four villages were used to examine the type of providers that were sought for treatment, differences of costs across different providers and inequity in total costs. In equity analysis, an asset based index was used to categorize the households into SES quartiles: least poor, poor, very poor and most poor. Principal components analysis (PCA) was used to generate the index [27] that was used to investigate the equity implications of the findings. Information on ownership of a radio, bicycle, motor car, grinding machine and motorcycle together with the weekly per capita cost of food was used to generate the SES index. Chi-square test was used to determine the SES differences of the key dependent variables. **Note: **120 Naira = 1 US$

### Ethical Clearance

This research was approved by the ethical clearance committee of the College of Medicine University of Nigeria, Enugu Campus Enugu State Nigeria.

## Results

Table 1 presents the descriptive characteristics of the respondents and their households. The table shows that most of the respondents were the household heads who were mostly married men without any formal education, except in Ahani where about 67.4% of them had some formal education. The average years of formal education was less than 5 years in the four villages and this is quite low. The table also shows that the average household size ranges from 3.7 residents in Enugu-akwu to 5.2 in Adu. The major source of income in the four villages was subsistence farming. However quite a number of the villagers also engaged in petty trading and very few of them were professionals or into big business.

**Table 1:** Socio-economic and demographic characteristics of respondents and their household

	Adu N = 299	Ahani N = 298	Amaetiti N = 300	Enugu-Akwu N = 300
Household head: n (%)	205 (68.6)	147 (49.3)	216 72.0	157 52.3
Male respondents: n (%)	124 (41.5)	109 (36.6)	131 (43.7)	116 38.7
Age: Mean (SD)	57.54 (58.0)	50.523 (14.77)	53.64 (13.98)	53.4 (16.3)
Had formal education: n (%)	143 (47.8)	201 (67.4)	111 (37.0)	144 (48.0
Years of formal education				
Mean (SD)	3.05 (4.30)	4.16 (4.26)	2.67 (4.10)	3.32 (4.12)
**Ever married?**				
0 = no: n (%)	37 (12.4)	29 (9.7)	25 (8.3)	47 (15.7)
1 = yes: n (%)	262 (87.6)	269 (90.3)	275 (91.7)	253 (84.3)
No of household residents				
Mean (SD)	5.18 (3.59)	3.85 (2.01)	4.94 (2.61)	3.70 (2.11)
**Occupation**				
Unemployed/housewives: n (%)	19 (6.4)	9 (3.0)	21 (7.0)	37 12.3
Farmers: n (%)	199 (66.6)	210 (70.5)	204 (68.0)	180 60.0
Petty traders/skilled labor: n (%)	50 (16.7)	42 (14.1)	29 (9.7)	48 16.0
Regular wage earner: n (%)	29 (9.7)	29 (9.7)	42 (14.0)	26 8.7
Professionals & big biz: n (%)	2 (0.7)	8 (2.7)	4 (1.3)	9 3.0

### Incidence of malaria and morbidity for Adult respondents and for children

Table 2 shows that the incidence of presumptive malaria for adults ranged from 29.5% to 46.5% in the four villages. The morbidity from malaria lasted from approximately 5 days in two of the villages to 6 days in the other two villages (Adu and Enugu-akwu) for adult malaria. Half of the adults that had malaria sought treatment after about two days. The table also shows that some of the children had malaria within one month of the survey, with the highest incidence being in Adu (34.8%). The mean ages of the ill children were generally above 5 years and the illness lasted for 3 to 5 days. As was in the case of adult malaria, treatment was sought for most of the ill children but just about an average of one day elapsed between the time the child became ill and the time treatment was sought. The results showed that in childhood malaria, someone had to stop work to care for the sick child and in majority of the villages; it was the adults who had to stop work for about 1 to 2 days to care for the sick child.

**Table 2:** Incidence of malaria and morbidity for respondents and children

	Adu N = 299	Ahani N = 298	Amaetiti N = 300	Enugu-Akwu N = 300
**Adult Respondents**				
No that had malaria in past month: n(%)	139 (46.5)	88 (29.5)	89 (29.7)	104 (34.7)
Number of days sick with malaria				
Mean (SD)	6.01 (6.29)	4.99 (5.81)	4.73 (3.93)	6.00 (5.81)
Treatment was sought: n (%)	131(94.24)	77 (97.5)	85 (95.51)	93 (89.42)
Days that elapsed before treatment was sought: Mean (SD)	1.86 (2.31)	1.47 (2.75)	1.64 (1.11)	1.46 (1.37)

**Children (less than 13 years)**				
No of households with incidence of childhood malaria: n (%)	104 (34.8)	36 (12.1)	38 (12.7)	53 (17.7)
Age of the ill child: Mean (SD)	7.47 (4.99)	6.42 (5.18)	5.32 (3.46)	6.29 (4.39)
No of days malaria lasted: Mean (SD)	4.68 (2.58)	3.64 (3.07)	3.65 (5.52)	4.36 (3.08)
Treatment was sought: n (%)	98 (94.2)	34 (94.4)	34 (89.5)	53 (100)
Days that elapsed before treatment was sought: Mean (SD)	1.25 (1.26)	1.11 (0.89)	1.50 (1.85)	1.34 (0.98)
Cases where someone stopped work to care for a child n(%)	45 (43.30%)	31 (86.11%)	31 (81.58%)	13 (24.53%)
Person that stopped work so as to care for the sick child				
Adult: n (%)	32 (30.77)	17 (47.22)	9 (23.68)	12 (22.64)
Teenager: n (%)	2 (1.92)	5 (13.89)	5 (13.16)	0 (0.0)
Child: n (%)	13 (12.5)	9 (25.0)	17 (44.74)	1 (1.89)
Days missed work to care for the child:				
Mean (SD)	1.56 (2.05)	1.97 (2.54)	2.47 (3.16)	0.57 (1.33)

There were no SES differences in occurrence of reported adult malaria. However, children belonging to the least poor households had greater incidence of presumptive malaria compared to children from the very poor and most poor households (p < .05). Similarly, there was no SES difference in the number of days that the adults and children were ill, with the exception of one of the villages (Enugu-Akwu) where the children in the least poor SES group were ill with malaria for much longer than the other SES groups (p < 0.05).

### Health seeking for malaria

The SES groups in the four villages had a similar pattern of health seeking for both adult and childhood malaria with the exception of adults in Ahani where the least poor households sought more treatment than the others and in Enugu-akwu, where treatment for childhood malaria was mostly sought by the least poor households. From the pooled data of the four villages, the findings show that for the 420 adults that had presumptive malaria, the highest proportion of 35.0% used patent medicine dealers, 17.4% used hospitals, 7.6% used home treatment, 9.5% used clinics, 10% used other sources of treatment such as herbalists and 9.5% did not seek any treatment. Similarly, in the case of childhood presumptive malaria, for the 231 cases, treatment was sought for 51.7% of the cases in patent medicine dealers, 9.1% used hospitals, 7.0% used home treatment, 9.1% used clinics and 2.2% used other providers.

### Average monthly expenditures for the treatment of malaria

Table 3 shows that the average expenditure to treat an episode of malaria for those that had the disease ranged from as low as 131 Naira ($1.09) in Amaetiti to as high as 348 Naira ($2.4) in Adu. The average transportation cost to receive treatment for malaria was highest in Adu at about 46 Naira (less than $1). Most of the respondents recovered after the first treatment of malaria. The Table shows that the expenditures to treat childhood malaria was lower than that for adults in the four villages The highest average cost for the treatment of childhood malaria was in Amaetiti at 282 Naira ($ 2.0). The average transportation costs for a child to receive treatment in the four villages were all less than 20 Naira (less than $1) per visit. People from Ahani and Amaetiti traveled the longest distances to receive treatment. In Ahani, only about 30% of the children recovered from the first treatment they received while in the other three villages more than 50% of the children recovered. There were no socio-economic differentials in the expenditures to treat both adult and childhood malaria in the four villages, with the exception of adult malaria in Enugu-akwu where the least poor households incurred the highest expenditure of 354.1 Naira ($ 2.5). Similarly, there were also no socio-economic differentials in the expenditures on transportation for both adult and childhood malaria, with the exception of childhood malaria in Amaetiti and Enugu-akwu, where the least poor households incurred the highest expenditures on transportation at 76.52 Naira in Enugu-akwu and 34.8 Naira in Adu. There were no SES differences concerning whether the adult respondents recovered but children belonging to the least poor SES groups recovered faster after an episode of malaria in comparison to those belonging to other SES groups.

**Table 3:** Average monthly expenditures for the treatment of malaria for Adults and Children

	Adu	Ahani	Amaetiti	Enugu-Akwu
**Adult Respondents**	**N = 139**	**N = 88**	**N = 89**	**N = 104**
Cost of treatment: Mean (SD)	347.49 (637.42)	236.88 (523.96)	131.18 (165.61)	263.7 (343.6)
Cost of transport: Mean (SD)	45.96 (112.19)	39.67 (92.46)	31.69 (72.66)	25.5 (87.2)
Did respondent recover: n (%)	101 (72.66%)	64 (72.73%)	75 (84.27%)	80 (76.92%)

**Children (less than 13 years)**	**N = 104**	**N = 36**	**N = 38**	**N = 53**
Cost of treatment: Mean (SD)	176.3 (406.9)	97.78 (186.58)	281.77 (879.04)	190.9 (378.5)
Transportation cost: Mean (SD)	15.5 (38.1)	17.48 (37.14)	17.06 (68.20)	16.4 (42.9)
Did child recover: n (%)	58 (55.77%)	13 (36.11%)	21 (55.26%)	33 (61.27)

**Note: **120 Naira = 1 US$

The pooled data from the 4 villages showed that there were no SES differences in total costs for both adult and childhood malaria (p > 0.05). Comparing total costs across different providers incurred by adults, the least total costs of 140.0 Naira (SD 277.0) were incurred for home treatment and the highest total costs of 870.3 Naira (SD 941.1) were incurred in hospitals. The average costs for other providers were: 210.6 Naira (SD 227.2) for health clinics; 199.7 Naira (SD 317.5) for patent medicine dealers; and 185.8 (SD 204.5) for community health workers. A similar pattern was also found for children.

### Expenditures to prevent malaria and ownership of mosquito nets

The cost of preventing malaria in the four villages was generally low although households in Amaetiti spent more on malaria preventive measures than the other villages (Table 4). A greater percentage of the households in the four villages did not own an untreated bed net, (less than 5% of all households owned an untreated net). Similarly ownership of insecticide treated bed nets (ITN) was also very low and the highest percentage of households in the villages with ITN was found in Ahani (10.4%).

**Table 4:** Average monthly expenditures for the prevention of malaria and ownership of bednets in the four villages

	Adu	Ahani	Amaetiti	Enugu-akwu
Expenditures for preventing mosquito nuisance in past month: Mean (SD)	80.32 (230.17)	11.17 (52.12)	112.87 (245.63)	28.2 (129.5)
Owns an untreated net: n(%)	20 (6.7)	11 (3.7)	6 (2.0)	10 (3.0)
Owns an ITN: n (%)	7 (2.3)	32 (10.4)	9 (3.0)	15 (5.0)

**Note: **120 Naira = 1 US$

Table 5 shows that there were SES differences in prevention of malaria, with the most-poor SES group being worse-off. Expenditures to prevent malaria increased as SES group increased and the finding was statistically significant in three of the villages. Also, it was observed that there was a likely hood that half of the higher SES groups would own both treated and untreated mosquito nets in the villages except in the case of Amaetiti and Enugu-Akwu for untreated nets and Ahani and Amaetiti for ITN which were not statistically significant.

**Table 5:** SES differences in expenditures to prevent malaria and ownership of mosquito nets

	Adu	Ahani	Amaetiti	Enugu-akwu
Cost of prevention				
Q1 = most-poor (SD)	12.93 (38.38)	8.15 (40.12)	45.33 (123.6)	7.33 (30.55)
Q2 = very poor (SD)	61.51 (171.03)	7.67 (42.48)	169.33 (261.7)	42.47 (198.15)
Q3 = poor (SD)	71.01 (210.15)	5.54 (41.02)	64.00 (146.6)	36.13 (133.78)
Q4 = least poor (SD)	177.23 (354.8)	23.42(75.52)	172.8 (353.0)	26.67 (94.84)
Chi^2^	19.08	9.14	19.44	1.63
(p-value)	0.0003	0.03	0.0002	0.65

Ownership of untreated bed-nets				
Q1 = most-poor (%)	0 (0)	0 (0)	1 (1.3)	0 (0)
Q2 = very poor (%)	3 (4)	0 (0)	3 (4)	2 (2.7)
Q3 = poor (%)	4 (5.3)	3 (4)	1 (1.3)	3 (4)
Q4 = least poor (%)	13 (17.6)	8 (10.8)	1 (1.3)	4 (5.3)
Chi2	20.52	16.33	2.04	4.01
(p-value)	0.0001	0.001	0.56	0.26

Ownership of insecticide-treated nets				
Q1 = most-poor (%)	0 (0)	3 (4)	1 (1.3)	1 (1.3)
Q2 = very poor (%)	1 (1.3)	7 (9.3)	2 (2.7)	1 (1.3)
Q3 = poor (%)	1 (1.3)	9 (12)	3 (4)	4 (5.3)
Q4 = least poor (%)	5 (6.8)	12 (16.2)	3 (4)	9 (12)
Chi2	8.78	6.32	1.26	12.00
(p-value)	0.03	0.09	0.73	0.007

**Note: **120 Naira = 1 US$

## Discussion

The findings show that the different SES groups had almost equal exposure to malaria and suffer similar morbidity patterns when they contract the disease. They also have similar health seeking patterns. However, it is possible that the better-off SES group has an increased perception of disease occurrence, and hence reported malaria occurrence is more amongst them than the worse-off SES. Nonetheless, the finding supports the argument that the relationship between incidence of malaria and wealth distribution is not strong after controlling for confounding factors [25]. It was also found that the better-off SES more than the worse-off SES sought treatment once they had malaria.

It is surprising that a larger proportion of adults reported malaria in the last one month compared to children, who are known to be more vulnerable to malaria. The reasons for this apparent anomaly were not investigated in the study but should be an area for future studies where similar findings occur. However, one speculation was that since male household heads were the majority of the respondents instead of their wives, who are usually the major household care givers, the respondents may have under-reported the occurrence of childhood malaria.

There was a shorter delay in seeking treatment for childhood malaria compared to adult malaria, which could explain the finding that the length of days that people were ill with malaria was more in adults compared to children. People are likely to be concerned more when a child is ill than when an adult is, thereby accounting for the quicker response period to seek care for childhood malaria as compared with adult malaria. This was also reflected in the finding that whilst some adults did not seek treatment, all the children were treated for malaria. The lower expenditure on treatment for childhood malaria compared to that of adults is explained by the fact that children require lower dosage of drugs than adults, especially when viewed from the finding that treatment was sought more for children in patent medicine dealers, whilst a higher proportionate use of hospitals was found for adults. However, the fact that a minority of the children recovered from the first treatment that they received in the four villages means that either the patients did not have malaria in the first place or that the treatments given were inappropriate in many cases. The latter may be the case when viewed from the findings that care was mostly sought from drug sellers (patent medicine dealers).

The findings showing only statistical significant SES differences in expenditures for prevention of malaria but not for treatment, means that the poorer socioeconomic groups potentially suffer more because a greater proportion of their financial resources are inevitably spent on the treatment and prevention of the disease when compared to their SES. This was very evident in one of the villages and is in line with the findings in another study where households at lower socio-economic levels spent greater shares of their income than better-off households [4]. Such expenditures by the most-poor and very-poor SES could be catastrophic as there is a growing evidence of households being pushed into poverty or forced into deeper poverty when faced with substantial medical expenses, particularly when combined with a loss of income due to ill health [28]. The indirect costs of malaria as deduced from the number of days lost due to malaria, especially because adults give up activities like going to work to care for children when they have malaria reduces the number of days they go to work [14] which further erodes the earning of households, with greater impact on the poorest SES that mostly depend on daily subsistence.

The findings show that the poorer SES spent less money on prevention of malaria and owned fewer mosquito nets. These low level of expenditures implicitly mean that the poor will be more exposed to mosquito bites and hence malaria. Thus as argued in another paper, although malaria may adversely affect economic activity and lead to poverty, it is also possible that the poor are less able to protect themselves from malaria and less able to seek effective treatment and therefore experience greater morbidity from the disease [4]. However, future studies should examine the inter-relationships of some of the variables and link them with the burden of malaria. For example, it will be interesting to determine whether those who have higher transportation costs for malaria are more likely to suffer greater morbidity of even die from malaria. Another area for future studies is to investigate the reasons for the low levels of use of appropriate malaria treatment services by the people.

## Conclusion

All in all, the paper shows that all SES groups suffer equal exposures and burden of malaria but the better-off SES spend higher amounts of money to prevent malaria and also possess more mosquito nets compared to poorer households. The finding that highest total costs were incurred in hospitals suggests that strategies should be developed that will ensure that the poor pay relatively lower amounts of money for the treatment of malaria there and that will encourage the greater use of hospitals compared to patent medicine dealers where the quality of treatment is usually sub-optimal. Strategies are also needed that will ensure that people have improved access and ownership of malaria preventive tools especially insecticide-treated nets, so as to substantially decrease the burden of the disease especially amongst the most-poor households. The effects of these interventions will in the long-run lead to improved productivity and well-being of all the households.

## Competing interests

The authors declare that they have no competing interests.

## Authors' contributions

OO conceived the study. All the authors participated in data collection and analysis. NU wrote the manuscript with input from all the authors

## Pre-publication history

The pre-publication history for this paper can be accessed here:

http://www.biomedcentral.com/1472-698X/9/21/prepub
